# Health risk assessment of disinfection by-products in drinking water on children and adolescents aged 6–17 in Ningbo city, China

**DOI:** 10.3389/fpubh.2026.1762519

**Published:** 2026-03-20

**Authors:** Xuefei Zhao, Qun Zhang, Bijun Shi, Dandan Zhang

**Affiliations:** Ningbo Municipal Center for Disease Control and Prevention, Ningbo, China

**Keywords:** Carcinogenic risk, disinfection by-products, drinking water, health risk assessment, non-carcinogenic risk

## Abstract

**Background:**

Drinking water disinfection generates disinfection by-products (DBPs) with adverse health impacts. Despite regulatory measures to limit DBPs levels, uncertainties remain about cumulative risks from complex DBPs mixtures, especially for children and adolescents. This study used centralized water supply quality monitoring data from Ningbo to assess health risks of DBPs via drinking water ingestion for children and adolescents aged 6–17.

**Methods:**

886 water samples were collected from 69 waterworks in Ningbo. Six DBPs (trichloromethane, dibromochloromethane, bromodichloromethane, tribromomethane, dichloroacetic acid, and trichloroacetic acid) were analyzed in accordance with the Chinese Standard Examination Methods for Drinking Water (GB/T 5750-2023). The US EPA health risk assessment model was used to evaluate the carcinogenic and non-carcinogenic risks of DBPs via drinking water ingestion in children and adolescents aged 6–17 years (divided into four age groups).

**Results:**

Across the four age groups, the median carcinogenic risks of the six DBPs were all below 10^−4^, with the total median carcinogenic risk ranging from 27.487 × 10^−6^ to 75.997 × 10^−6^. Meanwhile, the median hazard quotients for the four age groups were all <1, and the total hazard quotient ranged from 4.181 × 10^−2^ to 6.422 × 10^−2^.

**Conclusion:**

The health risks associated with the six DBPs via drinking water ingestion is acceptable for children and adolescents aged 6–17 in Ningbo. To further reduce potential risks, measures such as upgrading water distribution systems, enhancing source water treatment, and optimizing disinfectant type selection are recommended.

## Introduction

Safe drinking water is fundamental to public health, and disinfection is a critical step in ensuring its microbiological safety by eliminating pathogens such as bacteria, viruses, and protozoa (1). However, disinfection processes that commonly using chlorine, chloramine, or ozone can react with natural organic matter (OM), inorganic compounds, and other precursors present in water, forming a diverse group of disinfection by-products (DBPs) (2). These DBPs, including trihalomethanes (THMs), haloacetic acids (HAAs), and haloacetonitriles (HANs), halonitromethanes (HNMs), halogenated acetamides (HAMs), have raised widespread concerns due to their potential toxicity (3, 4).

Children and adolescents are particularly vulnerable to environmental contaminants, including DBPs, due to their unique physiological characteristics: rapid growth and development, higher water intake relative to body weight, and immature detoxification systems (5, 6). Epidemiological and toxicological studies have linked chronic exposure to DBPs with adverse health outcomes in this population, such as developmental issues, respiratory problems, and an increased risk of certain cancers (7–9). Despite regulatory efforts to limit DBPs concentrations in drinking water, uncertainties remain regarding the cumulative risks posed by complex DBPs mixtures, especially for sensitive groups like children and adolescents.

In China, the significance of DBPs control in drinking water has been increasingly emphasized, as reflected in a comprehensive framework of regulatory standards, technological innovation, and scientific research. Specifically, the Chinese national standards for drinking water quality (GB 5749-2022) (10) was revised in 2022 to address both the potential adverse health effects of DBPs exposure and the rising demand for safe drinking water. This standard incorporates regulatory adjustments for four THMs [trichloromethane (TCM), dibromochloromethane (DBCM), bromodichloromethane (BDCM), and bromoform (TBM)] and two HAAs [dichloroacetic acid (DCAA) and trichloroacetic acid (TCAA)]. notably, all indicators except TCM have been reclassified from expanded indices to regular indices. Epidemiological investigations have begun to examine associations between DBPs exposure and health outcomes in Chinese populations, providing foundational data for risk assessment models tailored to local contexts (11–13). However, most studies focus on adults or treat children under 18 years of age as a single sensitive group when assessing DBPs-related risks, with few dividing them into different age subgroups (14, 15). Given the significant differences in DBPs exposure features across different age groups of children, it is both necessary and of great significance to conduct age-specific health risk assessments of DBPs in drinking water for children.

Considering the differences in DBPs-related health risks and targeted prevention strategies across different age groups of children, this study adopts the health risk assessment model proposed by the U.S. Environmental Protection Agency (US EPA) and uses centralized water supply quality monitoring data from Ningbo to conduct a age-specific health risk assessment of children exposed to DBPs via drinking water ingestion. It aims to identify the primary DBP pollutants affecting drinking water safety and the most sensitive age groups of children, thereby providing a scientific basis for drinking water safety and water safety management and targeted DBPs risk mitigation strategies for children of different ages in Ningbo.

## Materials and methods

### Study area

This study was conducted in Ningbo (120.55°-122.16°E, 28.51°-30.33°N), a coastal city located in the middle of China's mainland coastline and the southern part of Yangze River Delta, bordering the East China Sea. Ningbo is a water-scarce city, with a per capita water resource possession of approximately 855 cubic meters, which is less than 50% of the national average and 60% of Zhejiang Province's average.

### Sample collection

Based on the distribution of urban and rural centralized water supply networks in Ningbo, the Ningbo Center for Disease Control and Prevention (CDC) has established water quality monitoring points covering all sub-districts and towns in the city. Stratified random sampling was adopted for monitoring point layout. For urban areas, including Ningbo's main urban area and sub-districts/towns within county-level urban areas, at least one terminal water monitoring point and one secondary water supply monitoring point were randomly set up per 20,000 people supplied by each waterworks. Sampling was conducted once in the dry season (March to April) and once in the wet season (July to August), with one sample collected each time from each waterworks' finished water, terminal water monitoring points and secondary water supply monitoring points. For rural areas, defined as sub-districts and towns not included in the aforementioned urban areas, 1–5 terminal water monitoring points were randomly deployed in each area based on the local waterworks' supply population and capacity. Sampling was also conducted once in both the dry and wet seasons, with one sample collected each time from each waterworks' finished water and terminal water monitoring points.

A total of 886 water samples were collected from 69 waterworks, including 442 samples in the dry season and 444 in the wet season; 138 samples of finished water, 550 of terminal water and 198 of secondary water supply; 854 samples of surface water and 32 of ground water. There were 718 samples disinfected with sodium hypochlorite, 112 with liquid chlorine, 52 with composite chlorine dioxide (with chlorine dioxide as the main component and chlorine as the auxiliary component), and four with bleaching powder.

Finished water refers to the water from centralized water supply units that, after completing the treatment process, is about to enter the distribution pipe network. Terminal water refers to the water that reaches users' taps after the finished water is transported through the distribution pipe network. Secondary Water Supply is a system that stores, pressurizes and redistributes water (treated by waterworks to municipal pipeline terminals) via dedicated tanks, reservoirs and pumps, to supply high-rises or areas with inadequate primary network pressure.

### Sample analysis

Six DBPs were analyzed in the water samples, including TCM, DBCM, BDCM, TBM, DCAA and TCAA. Meanwhile, water quality parameters (WQPs) including pH, permanganate index (PI, calculated as O_2_) and free chlorine (FC), were determined in the water samples, and the collected data were used to explore their correlations with the formation of the six DBPs.

The collection, preservation, and analysis of DBPs were conducted in accordance with the Chinese Standard Examination Methods for Drinking Water (GB/T 5750-2023) (16). Samples were placed in brown glass bottles to avoid sunlight exposure, with filling continued until the bottles overflowed. Hydrochloric acid was used to adjust the pH ≤ 2. Water samples were stored and transported at 4 °C.

According to GB/T 5750-2023, headspace capillary column gas chromatography method was used to determine TCM, DBCM, BDCM and TBM, while liquid-liquid extraction derivatization gas chromatography method was used to determine DCAA and TCAA. The limits of detection (LODs) of the analytical methods are listed in Table 1, and values reported below the LOD were imputed as LOD/2 during data processing (11). Additionally, pH was determined on-site using the glass electrode method, PI was determined by acidic potassium permanganate titration method, and FC was determined on-site using the N,N-diethyl-p-phenylenediamine (DPD) method.

**Table 1:** Toxicology coefficients of the DBPs (11, 19–21).

**DBPs**	**LOD (ug/L)**	**SF (mg/kg·day)^−1^**	**RfD (mg/kg·day)**
TCM	0.032	0.031	0.010
DBCM	0.015	0.084	0.020
BDCM	0.016	0.062	0.020
TBM	0.041	0.0079	0.020
DCAA	2.000	0.050	0.004
TCAA	1.000	0.070	0.020

### Health risk assessment

The health risk assessment model proposed by the US EPA was adopted to evaluate the current carcinogenic and non-carcinogenic risks of the DBPs via drinking water ingestion (17). The values of population exposure parameters, including body weight (BW) and daily drinking water ingestion rate (IR), were derived from the *Exposure Factors Handbook of Chinese Population* (6–17 years) (6, 18). According to this handbook, children aged 6–17 years in this study were divided into four age groups, and the health risks for each age group were calculated. The slope factor (SF) and reference dose (RfD) of the DBPs in drinking water were obtained from the Integrated Risk Information System (IRIS) and Risk Assessment Information System (RAIS) (11, 19–21). Relevant variables and parameters are detailed in Tables 1, 2.

**Table 2:** Exposure parameters for health risk assessment of drinking water contaminants in children aged 6–17 in Ningbo City (6, 18).

**Population exposure parameters**	**Gender**	Age group (year)			
		**6–8**	**9–11**	**12–14**	**15–17**
IR (L/d)	male	0.690	0.717	0.794	0.885
	female	0.665	0.701	0.755	0.915
	total	0.679	0.709	0.775	0.899
BW (kg)	Male	27.2	35.8	47.0	58.6
	female	26.2	33.2	44.8	49.8
	Total	26.7	34.6	45.9	54.3

### Carcinogenic risk assessment

Carcinogenic risk (CR) is typically expressed as a numerical risk value. Generally, a CR lower than 1 × 10^−6^ indicates negligible risk to human health, whereas a CR greater than 1 × 10^−4^ signifies substantial potential carcinogenic risk. A CR ranging from 1 × 10^−6^ to 1 × 10^−4^ is deemed acceptable for human health, though it carries inherent potential risks that require enhanced risk monitoring (22).

Additionally, in accordance with the US EPA guidelines on heightened cancer susceptibility during early life, age-dependent adjustment factors (ADAFs) were incorporated into the carcinogenic risk calculation (23, 24), and the weighted average method was employed for calculating the CR of the 15–17 age group.

In cases involving multiple pollutants, the total carcinogenic risk (CR_t_) is calculated as the sum of the individual risks posed by each pollutant. The formulas are as follows:


$$\begin{array}{ccc} ADD & = & \left(C\times IR\times EF\times ED\right)/\left(BW\times AT\right)\; \end{array}$$ (1)

$$\begin{array}{ccc} CR & = & SF\times ADD\times ADAFs\; \end{array}$$ (2)

$$\begin{array}{ccc} CR_{15-17} & = & \left(CR_{15}\times ED_{15}+CR_{16-17}\times ED_{16-17}\right)/ED_{15-17} \end{array}$$ (3)

$$\begin{array}{ccc} {CR}_{t} & = & \sum CR_{i} \end{array}$$ (4)

Among them, *ADD* is the average daily potential dose of the DBPs via drinking water ingestion; *C* is the concentration of DBPs in drinking water; *IR* is the daily drinking water ingestion rate; *EF* is the exposure frequency (365 days·year^−1^); *ED* is the exposure duration, set as 3 years for the 6–8, 9–11, and 12–14 age groups; for the 15–17 age group, a subdivision is made at the age of 16: *ED* is 1 year for 15-year-olds and 2 years for 16–17-year-olds, with a total *ED* of 3 years for the entire age group; *BW* is the average body weight; *AT* is the averaging time (*ED*× 365 days); *ADAFs* are set as three for the 6–15-year-olds and one for the 16–17-year-olds.

### Non-carcinogenic risk assessment

Non-carcinogenic risks are assessed using the hazard quotient (HQ). Typically, an HQ value below one signifies a low non-carcinogenic risk, while an HQ of one or higher indicates a high non-carcinogenic risk. The HQ for each component is calculated as the ratio of the average daily dose (ADD) to the reference dose, as follows:


$$\begin{array}{ccc} HQ & = & ADD/RfD \end{array}$$ (5)

$$\begin{array}{ccc} HQ_{t} & = & \sum HQ_{i} \end{array}$$ (6)

### Statistical analysis

Statistical analyses were performed using R software (version 4.3.1) and Excel 2007. Continuous data with a non-normal distribution were expressed as median and interquartile range [*Median (Q*_*R*_*)*]. Spearman's correlation analysis was used to explore the relationship between the formation of DPBs and WQPs. The Mann-Whitney *U*-test was used for comparisons between two groups, while the Kruskal–Wallis *H*-test and the Jonckheere–Terpstra test were employed for comparisons among multiple groups. Differences were considered statistically significant at *p* < 0.05.

## Results

### Presence of DBPs and WQPs in drinking water

As shown in Table 3, the detection rates of TCM, DBCM, BDCM, TBM, DCAA and TCAA ranged from 35.10 to 98.98% among 886 water samples. Meanwhile, PI and FC were detected in all water samples, with median concentrations of 0.69 and 0.26 mg/L, respectively. The pH values ranged from 6.50 to 8.46, with a median value of 7.20.

**Table 3:** Detecting rates of the DBPs and WQPs in drinking water samples.

**DBPs and WQPs**	Concentration (mg/L)		**Number of samples above detection**	**Detection rate (%)**
	**Range**	***Median (Q**_*R*_ **)***		
TCM	ND^a^-5.030 × 10^−2^	1.100 × 10^−2^(1.091 × 10^−2^)	877	98.98
DBCM	ND−1.130 × 10^−2^	0.130 × 10^−2^(0.135 × 10^−2^)	810	91.42
BDCM	ND−1.770 × 10^−2^	0.469 × 10^−2^ (0.314 × 10^−2^)	865	97.63
TBM	ND−1.100 × 10^−2^	ND (0.008 × 10^−2^)	311	35.10
DCAA	ND−4.75 × 10^−2^	0.44 × 10^−2^ (1.00 × 10^−2^)	471	53.16
TCAA	ND−4.40 × 10^−2^	ND (0.65 × 10^−2^)	336	37.92
PI	0.20–2.49	0.69 (0.46)	886	100.00
FC	0.01–0.88	0.26 (0.30)	886	100.00

^a^ND (Non-detect) indicates below the LOD.

Table 4 presents the distribution of the DBPs and WQPs across different sampling periods and water supply stages.

**Table 4:** Distribution of the DBPs and WQPs across water supply stages and sampling periods ( × 10^−2^, mg/L, pH dimensionless).

**DBPs and WQPs**	**Sampling period**	Finished water		Terminal water		Secondary water supply		***H^*a*^***	***p***	***Z^*b*^***	***p***
		***M***	***Q**_*R*_*	***M***	***Q**_*R*_*	***M***	***Q**_*R*_*				
TCM	Dry season	0.570	0.708	0.941	0.734	1.100	1.032	32.492	<0.001	5.555	<0.001
	Wet season	1.095	1.293	1.437	1.200	1.700	1.270	18.696	<0.001	4.293	<0.001
	*Z*	−3.103		−6.360		−4.059					
	*P*	0.002		<0.001		<0.001					
DBCM	Dry season	0.106	0.182	0.121	0.160	0.140	0.110	1.464	0.481	1.112	0.266
	Wet season	0.100	0.163	0.140	0.140	0.138	0.120	4.166	0.125	1.037	0.300
	*Z*	−0.153		−2.167		−0.110					
	*P*	0.878		0.030		0.912					
BDCM	Dry season	0.348	0.306	0.440	0.350	0.480	0.320	19.492	<0.001	4.269	<0.001
	Wet season	0.445	0.370	0.521	0.300	0.562	0.319	7.599	0.022	2.494	0.013
	*Z*	−1.945		−2.673		−1.021					
	*P*	0.052		0.008		0.307					
TBM	Dry season	0.002	0.000	0.002	0.000	0.002	0.000	14.381	0.001	3.150	0.002
	Wet season	0.015	0.298	0.010	0.298	0.008	0.298	0.167	0.920	−0.257	0.797
	*Z*	−8.867		−18.092		−9.143					
	*P*	<0.001		<0.001		<0.001					
DCAA	Dry season	0.98	1.06	0.54	1.00	0.50	1.10	1.828	0.401	−1.043	0.297
	Wet season	0.10	0.57	0.10	1.14	0.10	1.14	4.206	0.122	1.958	0.0503
	*Z*	−3.925		−3.398		−0.929					
	*P*	<0.001		<0.001		0.353					
TCAA	Dry season	0.05	0.00	0.05	0.55	0.05	0.63	11.316	0.003	3.292	0.001
	Wet season	0.05	0.84	0.05	0.96	0.05	0.72	1.502	0.472	−0.655	0.513
	*Z*	−3.035		−3.834		−0.047					
	*P*	0.002		<0.001		0.962					
pH	Dry season	7.07	0.54	7.12	0.39	7.23	0.27	17.608	<0.001	3.871	<0.001
	Wet season	7.20	0.61	7.20	0.51	7.27	0.29	2.580	0.275	1.317	0.188
	*Z*	−0.931		−1.892		−0.409					
	*P*	0.352		0.059		0.682					
PI	Dry season	60.00	88.00	60.00	25.00	66.00	19.00	5.371	0.068	1.273	0.203
	Wet season	88.50	52.00	80.00	53.00	80.00	62.00	2.602	0.272	−0.536	0.592
	*Z*	−3.157		−7.589		−3.911					
	*P*	0.002		<0.001		<0.001					
FC	Dry season	50.00	20.00	25.00	29.00	22.00	23.00	77.326	<0.001	−7.523	<0.001
	Wet season	50.50	24.00	21.00	29.00	20.00	18.00	83.180	<0.001	−7.222	<0.001
	*Z*	−0.688		−2.097		−0.782					
	*P*	0.491		0.036		0.434					

^*a*^Kruskal–Wallis H-test.

^*b*^Jonckheere–Terpstra test.

For DBPs, TCM showed significant inter-stage differences in both seasons, with concentrations gradually increasing from finished water to terminal water and secondary water supply, and all stages had higher concentrations in wet season. DBCM showed no significant inter-stage variations, but terminal water had significant higher concentrations in the wet season. BDCM displayed significant inter-stage differences in both seasons (concentrations rising with supply stages), terminal water had higher wet-season concentrations, while finished and secondary water supply showed no seasonal variations. TBM had significant inter-stage differences in the dry season, and all stages had higher concentrations in the wet season concentrations. DCAA had no inter-stage variations, with lower wet-season concentrations in finished and terminal water. TCAA showed significant inter-stage differences in the dry season, with higher wet-season concentrations in finished and terminal water.

For WQPs, pH showed significant inter-stage differences in the dry season (with median values increasing with supply stages) but not in the wet season, with no seasonal differences across all stages. PI had no inter-stage differences but significantly higher levels in the wet season. FC showed significant inter-stage differences in both seasons (finished water concentrations were significantly higher than terminal and secondary water supply), and only terminal water had significant seasonal differences.

### Health risk of DBPs

As shown in Tables 5, 6, across the four age groups, the median CR values for TBM and TCAA were all below 10^−6^ (Equation 1–3), while those of TCM, DBCM, BDCM and DCAA were all below 10^−4^. The median CR_t_ values for the four age groups were 75.997, 61.237, 50.458 and 27.487 × 10^−6^, respectively (Equation 4). All median HQ values were below 1 (Equation 1, 5), with the corresponding median HQ_t_ values of 6.422, 5.175, 4.264 and 4.181 × 10^−2^ (Equation 6). Both carcinogenic and non-carcinogenic risks of the six DBPs differed significantly among age groups, showing a significant decreasing trend with increasing age. Gender-stratified analysis revealed that females had significantly higher risks than males in most subgroups: 6–8 years (TBM, DCAA, TCAA), 9–11 years (BDCM, TBM, DCAA, TCAA), and 15–17 years (all six DBPs). In contrast, males had significantly higher risks than females for TBM, DCAA, and TCAA in the 12–14-year group.

**Table 5:** Carcinogenic risks of the DBPs via drinking water ingestion ( × 10^−6^).

**DBPs**	**Gender**	Age group (year)								***H***	***p***	***Z***	***p***
	6–8		9–11		12–14		15–17					
		***M***	***Q**_*R*_*	***M***	***Q**_*R*_*	***M***	***Q**_*R*_*	***M***	***Q**_*R*_*				
TCM	male	25.951	25.745	20.489	20.326	17.282	17.145	8.583	8.515	674.005	<0.001	−25.495	<0.001
	female	25.965	25.760	21.600	21.429	17.240	17.104	10.442	10.359				
	total	26.016	25.809	20.963	20.796	17.273	17.136	9.409	9.335				
	*Z*	−0.126		−1.520		−0.147		−5.392					
	*P*	0.900		0.128		0.883		<0.001					
DBCM	male	8.310	8.630	6.561	6.814	5.534	5.747	2.749	2.854	465.384	<0.001	−21.972	<0.001
	female	8.315	8.635	6.917	7.183	5.521	5.733	3.344	3.473				
	total	8.331	8.652	6.713	6.971	5.531	5.744	3.013	3.129				
	*Z*	−0.784		−1.796		−0.796		−5.522					
	*P*	0.433		0.072		0.426		<0.001					
BDCM	male	22.129	14.826	17.471	11.706	14.737	9.874	7.319	4.90	962.319	<0.001	−31.063	<0.001
	female	22.141	14.835	18.419	12.341	14.701	9.850	8.904	5.966				
	total	22.184	14.863	17.875	11.976	14.729	9.868	8.024	5.376				
	*Z*	−0.277		−1.975		−0.310		−7.066					
	*P*	0.782		0.048		0.757		<0.001					
TBM	male	0.012	0.048	0.009	0.038	0.008	0.032	0.004	0.016	747.589	<0.001	−32.153	<0.001
	female	0.012	0.048	0.009	0.040	0.008	0.032	0.005	0.019				
	total	0.012	0.048	0.010	0.039	0.008	0.032	0.004	0.017				
	*Z*	−12.041		−16.505		−12.042		−16.616					
	*P*	<0.001		<0.001		<0.001		<0.001					
DCAA	male	16.743	38.052	13.218	30.042	11.150	25.340	5.538	12.58	457.340	<0.001	−24.032	<0.001
	female	16.752	38.073	13.936	31.672	11.123	25.279	6.737	15.311				
	total	16.784	38.146	13.524	30.737	11.144	25.327	6.071	13.797				
	*Z*	−8.410		−8.688		−8.410		−10.066					
	*P*	<0.001		<0.001		<0.001		<0.001					
TCAA	male	2.664	34.627	2.103	27.338	1.774	23.060	0.881	11.453	747.227	<0.001	−32.031	<0.001
	female	2.665	34.646	2.217	28.821	1.770	23.004	1.072	13.933				
	total	2.670	34.713	2.152	27.971	1.773	23.047	0.966	12.555				
	*Z*	−14.587		−14.884		−14.587		−15.810					
	*P*	<0.001		<0.001		<0.001		<0.001					

**Table 6:** Non-carcinogenic risks of the DBPs via drinking water ingestion ( × 10^−2^).

**DBPs**	**Gender**	Age group (year)								***H***	***p***	***Z***	***p***
		6–8		9–11		12–14		15–17					
		***M***	***Q**_*R*_*	***M***	***Q**_*R*_*	***M***	***Q**_*R*_*	***M***	***Q**_*R*_*				
TCM	male	2.790	2.768	2.203	2.186	1.858	1.844	1.661	1.648	173.415	<0.001	−12.645	<0.001
	female	2.792	2.770	2.323	2.304	1.854	1.839	2.021	2.005				
	total	2.797	2.775	2.254	2.236	1.857	1.843	1.821	1.807				
	*Z*	−0.126		−1.520		−0.147		−5.392					
	*P*	0.900		0.128		0.883		<0.001					
DBCM	male	0.165	0.171	0.130	0.135	0.110	0.114	0.098	0.102	159.705	<0.001	−12.438	<0.001
	female	0.165	0.171	0.137	0.143	0.110	0.114	0.119	0.124				
	total	0.165	0.172	0.133	0.138	0.110	0.114	0.108	0.112				
	*Z*	−0.784		−1.796		−0.796		−5.522					
	*P*	0.433		0.072		0.426		<0.001					
BDCM	male	0.595	0.399	0.470	0.315	0.396	0.265	0.354	0.237	287.204	<0.001	−16.334	<0.001
	female	0.595	0.399	0.495	0.332	0.395	0.265	0.431	0.289				
	total	0.596	0.400	0.481	0.322	0.396	0.265	0.388	0.260				
	*Z*	−0.277		−1.975		−0.310		−7.066					
	*P*	0.782		0.048		0.757		<0.001					
male	0.003	0.010	0.002	0.008	0.002	0.007	0.002	0.006	644.009	<0.001	−29.727	<0.001
TBM	female	0.003	0.010	0.002	0.008	0.002	0.007	0.002	0.007				
	total	0.003	0.010	0.002	0.008	0.002	0.007	0.002	0.007				
	*Z*	−12.041		−16.505		−12.042		−16.616					
	*P*	<0.001		<0.001		<0.001		<0.001					
DCAA	male	2.790	6.342	2.203	5.007	1.858	4.223	1.661	3.776	295.902	<0.001	−19.774	<0.001
	female	2.792	6.345	2.323	5.279	1.854	4.213	2.021	4.593				
	total	2.797	6.358	2.254	5.123	1.857	4.221	1.821	4.139				
	*Z*	−8.410		−8.688		−8.410		−10.066					
	*P*	<0.001		<0.001		<0.001		<0.001					
	male	0.063	0.824	0.050	0.651	0.042	0.549	0.038	0.491	637.156	<0.001	−29.759	<0.001
TCAA	female	0.063	0.825	0.053	0.686	0.042	0.548	0.046	0.597				
	total	0.064	0.826	0.051	0.666	0.042	0.549	0.041	0.538				
	*Z*	−14.587		−14.884		−14.587		−15.810					
	*P*	<0.001		<0.001		<0.001		<0.001					

### Characteristics of DBPs' health risks

**Figure 1:**
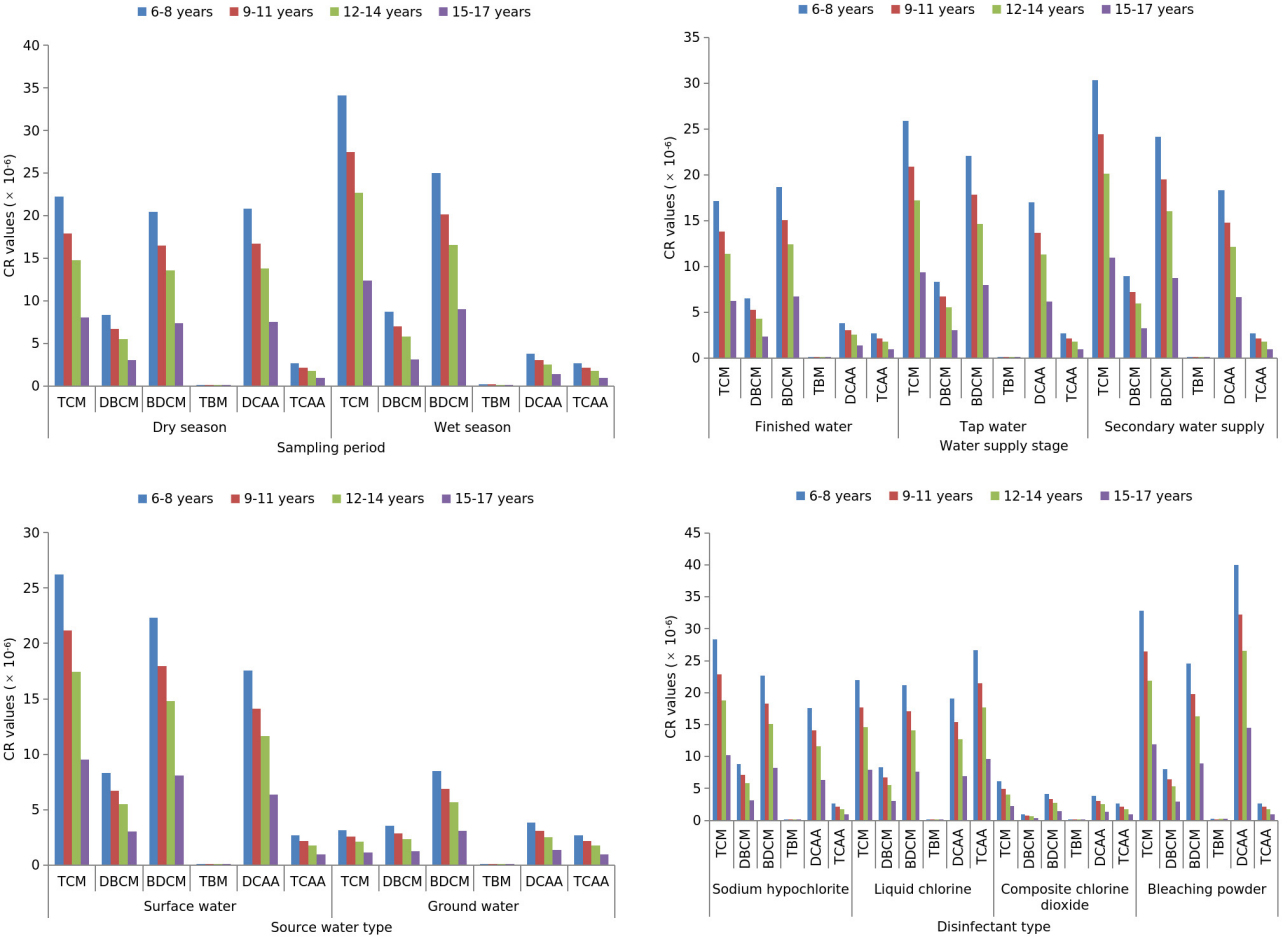
Stratified analysis of the carcinogenic risks of the DBPs via drinking water ingestion.

**Figure 2:**
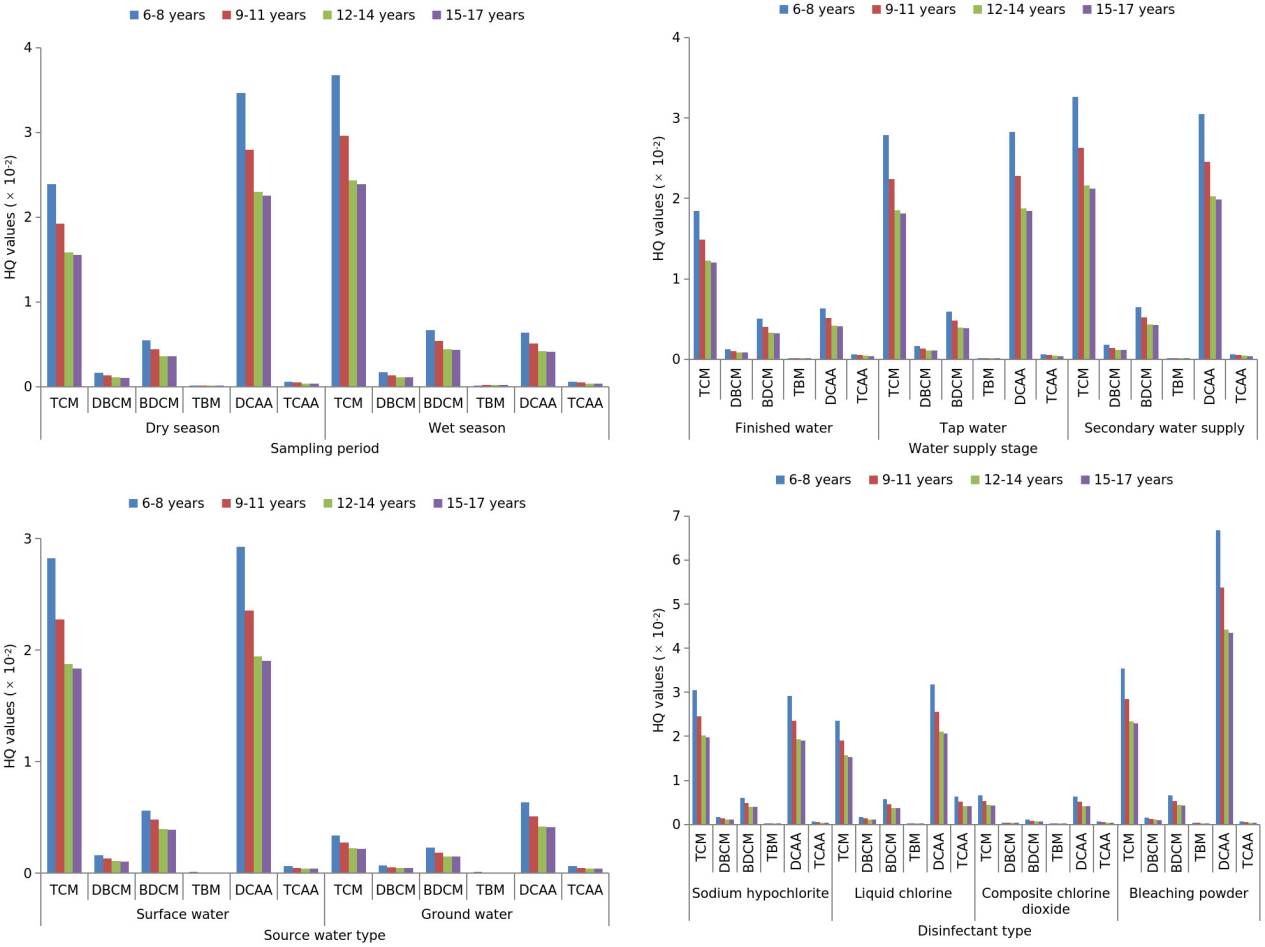
Stratified analysis of the non-carcinogenic risks of the DBPs via drinking water ingestion.

As shown in Figures 1, 2, TCM, DBCM, TBM and TCAA had significantly higher carcinogenic and non-carcinogenic risks in the wet season than in the dry season (*Z* =-7.969, −3.169, −22.084, −4.194; all *p* < 0.05). Conversely, DCAA had significantly lower risks in the wet season (*Z* = −4.662, *p* < 0.001). TCM and BDCM showed significant differences in both risk types among finished water, terminal water, and secondary water supply (*H* = 43.584, 23.563; all *p* < 0.001), with a significant increasing trend across supply stages (*Z* = 6.435, 4.541; all *p* < 0.001). Surface water had significantly higher carcinogenic and non-carcinogenic risks of TCM, BDCM, DCAA, and TCAA than groundwater (*Z* = −4.856, −4.916, −2.480, −2.276; all *p* < 0.05). Disinfectant type significantly affected both risk types for TCM, DBCM, BDCM, TBM, and TCAA (*H* = 76.785, 34.347, 63.204, 9.119, 19.169; all *p* < 0.05), with composite chlorine dioxide posing the lowest risks.

### Correlation matrix of DBPs and WQPs

Table 7 shows that TCM was positively correlated with BDCM (*r* = 0.806), DBCM (*r* = 0.202), DCAA (*r* = 0.204), and PI (*r* = 0.154), and negatively correlated with FC (*r* = −0.181). DBCM was positively correlated with BDCM (*r* = 0.579), pH (*r* = 0.316), and FC (*r* = 0.173), and negatively correlated with DCAA (*r* = −0.178) and TCAA (*r* = −0.130). BDCM had a positive correlated with pH (*r* = 0.084), and TBM was positively correlated with PI (*r* = 0.224) with no other significant associations. DCAA was positively correlated with TCAA (*r* = 0.168) and negatively correlated with pH (*r* = −0.241) and FC (*r* = −0.106). TCAA was negatively correlated with pH (*r* = −0.092), and had no significant correlation with FC (*r* = −0.057). pH was positively correlated with PI (*r* = 0.356) and negatively correlated with FC (*r* = −0.134). PI only correlated significantly with TCM and pH. FC was negatively correlated with TCM, DCAA, and pH, positively correlated with DBCM, and had no significant correlations with other parameters.

**Table 7:** Spearman's correlation matrix of analyzed parameters.

**DBPs and WQPs**	Correlations								
**TCM**	**DBCM**	**BDCM**	**TBM**	**DCAA**	**TCAA**	**pH**	**PI**	**FC**
TCM	1								
DBCM	0.202^**^	1							
BDCM	0.806^**^	0.579^**^	1						
TBM	0.134^**^	0.079^*^	0.018	1					
DCAA	0.204^**^	−0.178^**^	0.099^**^	−0.010	1				
TCAA	0.139^**^	−0.130^**^	0.026	0.110^**^	0.168^**^	1			
pH	0.037	0.316^**^	0.084^*^	−0.020	−0.241^**^	−0.092^**^	1		
PI	0.154^**^	0.050	0.052	0.224^**^	−0.115^**^	0.013	0.356^**^	1	
FC	−0.181^**^	0.173^**^	−0.030	−0.058	−0.106^**^	−0.057	−0.134^**^	−0.062	1

^**^Correlation is significant at the 0.01 level (two-tailed).

^*^Correlation is significant at the 0.05 level (two-tailed).

## Discussion

This study assessed the carcinogenic and non-carcinogenic risks of DBPs via drinking water ingestion for children and adolescents aged 6–17 in Ningbo City. Across four age groups, the median CR values of TBM and TCAA were <10^−6^, while those for TCM, DBCM, BDCM and DCAA were <10^−4^, with the median CR_t_ ranged from 27.487 to 75.997 × 10^−6^. All median HQ values were <1, with median HQ_t_ ranged from 4.181 to 6.422 × 10^−2^. These results indicate that DBP-related health risks for this population are within the acceptable range.

### Cross-study comparison of DBPs health risks

Several studies have evaluated DBPs-related health risks for Chinese children and adolescents. Lv et al. (25) reported that 1–18-year-olds in Hunan had cumulative DBPs carcinogenic risks (oral, dermal, inhalation exposure) of 1.15 × 10^−5^ to 2.56 × 10^−5^, with the highest risk in 1–2-year-olds. Du et al. (13) found that 1–18-year-olds across six provinces had cumulative DBPs carcinogenic risks exceeding 10^−4^ (oral, dermal, inhalation exposure), though the HI remained <1. Huang et al. (26) noted that combined exposure to arsenic and six DBPs (oral, dermal) resulted in total carcinogenic risks (TCR) of 1.4 × 10^−6^ to 2.04 × 10^−5^ for under−18s in four cities, with TCR and HI peaking in infants/toddlers and HI >1 in these age groups. Zhao et al. (6) observed similar TCR trends in Beijing and Guangzhou, with the highest risks in young children and HI >1 for 9-month−2-year-olds in Guangzhou. Our findings show lower DBPs-related risks, likely due to three factors. These factors are lower DBP concentrations in Ningbo's drinking water, exclusive focus on oral ingestion which excludes dermal and inhalation pathways that increase cumulative risk, and targeting 6 to 17 year olds which excludes the youngest and most vulnerable groups that drive higher risks in other studies.

### Age and gender differences in DBPs-related risks

Consistent with domestic studies (6, 13, 25), DBPs-related carcinogenic and non-carcinogenic risks decreased significantly with age. This is attributed to younger children having lower body weight and higher water intake relative to body weight, leading to higher per-unit exposure doses. Unlike previous studies, we observed age-specific gender differences. Females had higher risks for DCAA (6–8 years), BDCM and DCAA (9–11 years), and all six DBPs (15–17 years); Only 2–14-year-old males had higher DCAA risks. These differences may stem from water intake variations. Adolescent females may drink more water due to body image concerns or physical activity, while 12–14-year-old males may have higher water intake due to rapid growth and energy expenditure.

### Factors influencing DBPs-related risks

#### Seasonal variations

Seasonal risk differences were DBPs-specific. TCM, DBCM, TBM, and TCAA exhibited higher risks in the wet season, while DCAA showed the opposite trend. These observed seasonal disparities may be attributed to seasonal variations in water quality and the formation dynamics of DBPs (27–30). Wet season have higher temperatures and natural OM levels, accelerating chlorine-OM reactions and THMs formation. PI, an indicator reflecting the content of organic substances and oxidizable inorganic substances, was positively correlated with TCM (*r* = 0.154), confirming OM' s role as a THMs precursor. DCAA is sensitive to pH and temperature. High pH favors THMs formation but inhibits HAAs, and extreme heat may reduce DCAA yields. This is supported by our observation that DCAA was negatively correlated with pH and FC, with lower wet-season concentrations in finished and terminal water. Integrated strategies should be adopted to mitigate DBP formation risks, such as enhancing OM removal through optimized treatment processes, dynamically adjusting chlorine dosages and reducing water residence time.

#### Water supply stage dynamics

TCM and BDCM risks increased from finished water to terminal water to secondary supply, reflecting DBPs formation and accumulation in distribution systems (31). Conventional treatment controls initial DBP levels, but residual chlorine reacts with pipe-scale and biofilm-adsorbed OM in distribution networks to form additional DBPs (32). Secondary supply facilities such as rooftop tanks promote DBPs accumulation via prolonged storage. Prolonged storage reduces residual chlorine, which not only promotes bacterial proliferation but also necessitates rechlorination. The rechlorination process then reacts with remaining OM in water to generate more THMs (33). Additionally, sediment and rust buildup in secondary supply facilities enhances OM bioavailability, thereby further amplifying DBP-related health risks (34). This highlights the need to strengthen distribution system management including pipe cleaning, infrastructure upgrades and residual chlorine monitoring.

#### Source water type

Surface water posed higher DBPs-related risks than groundwater, as surface water such as rivers and lakes receives more anthropogenic and natural OM inputs including agricultural runoff, leaf litter and algal blooms that serve as DBPs precursors. Groundwater, filtered naturally through soil and aquifers, has lower OM content (35, 36). Given increasing reliance on surface water due to groundwater overexploitation, advanced OM removal technologies such as activated carbon adsorption and ozone oxidation are critical for reducing DBPs formation (37, 38).

#### Disinfectant type

Disinfectant type significantly affected DBPs risks. chlorine-based disinfectants including sodium hypochlorite, liquid chlorine and bleaching powder are more prone to forming TCM, DBCM, and BDCM, while composite chlorine dioxide or chlorine dioxide generates fewer of these DBPs (39, 40). These results support optimizing disinfectant selection to mitigation of targeted DBPs risks.

## Limitations and future directions

This study has several limitations. First, estimating DBPs concentrations as half the LOD may cause overestimation. Second, health risk assessment may be overestimated because this study used DBPs levels in unboiled water and ignores reductions from boiling (a common Chinese habit) (41). Third, only oral ingestion was considered as the exposure pathway. Skin contact and inhalation during showering were excluded, and these pathways affect total exposure. Further studies should address these gaps for more accurate risk assessment. Examples of such improvements include using DBPs data from boiled water and integrating multi-pathway exposure data.

## Conclusion

This study assessed the carcinogenic and non-carcinogenic risks associated with six DBPs (TCM, DBCM, BDCM, TBM, DCAA and TCAA) via drinking water ingestion for children and adolescents aged 6–17 years in Ningbo City. The results indicated that both types of risks were within the acceptable range. Significant variations were observed across age groups, gender, sampling periods, water supply stages, water sources, and disinfectant types. Specifically, relatively higher risks were associated with females in specific age groups, the wet season, secondary water supply systems, and surface water sources. These groups and scenarios should be prioritized for risk mitigation. To further reduce the DBPs-related risks, targeted management strategies are recommended, including upgrading water distribution systems, enhancing source water treatment processes, and optimizing disinfectant selection.

## Data Availability

The raw data supporting the conclusions of this article will be made available by the authors, without undue reservation.
